# Supplementary material for: Prognostic Role of Blood NETosis in the Progression of Head and Neck Cancer
Source: Cells. 2019 Aug 21;8(9):946. doi: 10.3390/cells8090946 (PMC6770876; doi:10.3390/cells8090946)
Supplement: Supplementary file 1 [file cells-08-00946-s001.pdf]

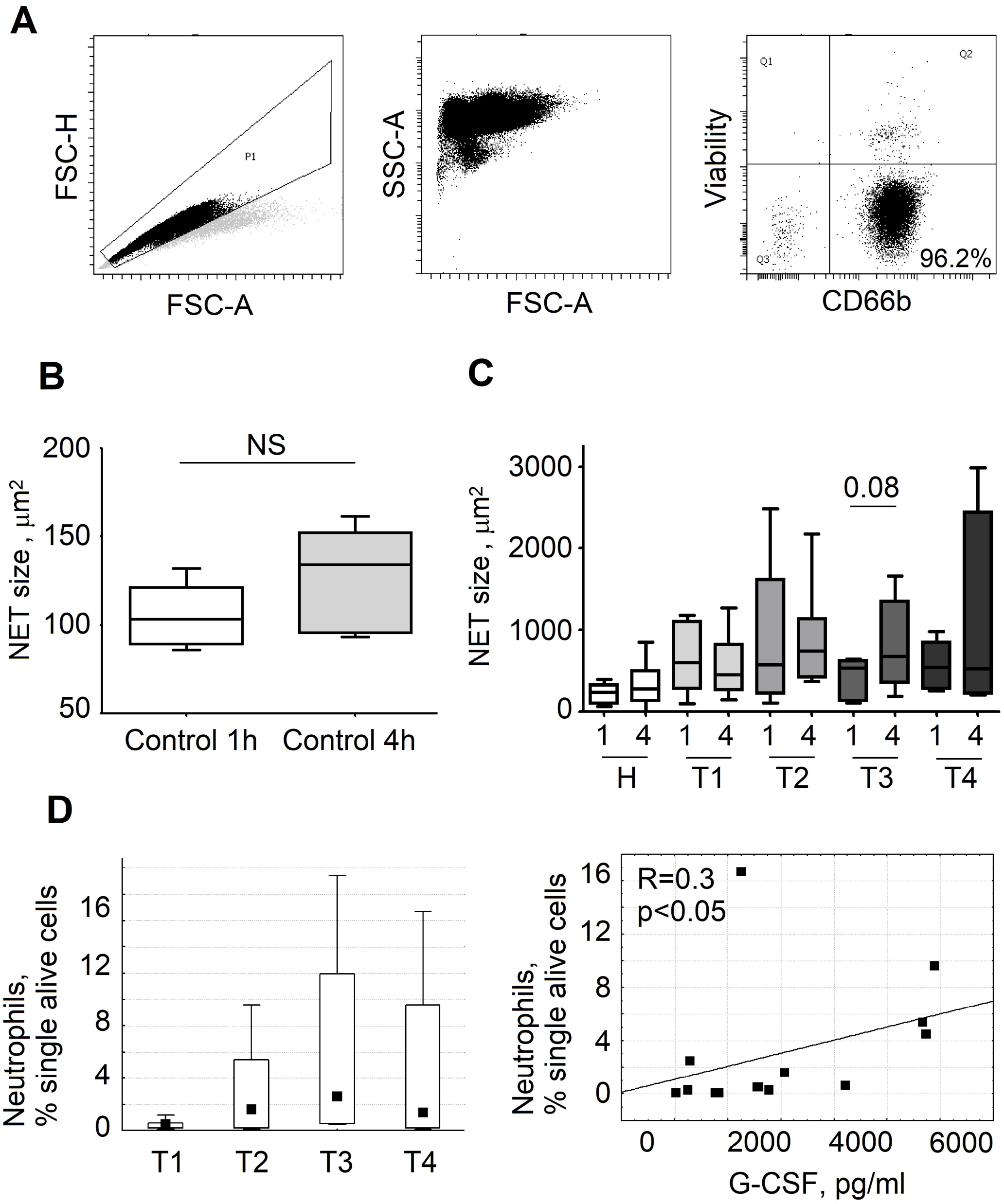

Figure S1. A. Gating strategy to determine the purity of isolated blood neutrophils (>95%). B. Comparison of NET formation by neutrophils from HNC patients in control conditions (with sterile medium) between 1 and 4 h. C. Comparison of *P. aeruginosa*-induced NET formation between 1 and 4 h in different T stages. H—healthy. D. Neutrophil content in HNC tissue and correlation of tumor-infiltrating neutrophils with G-CSF content in tumors.

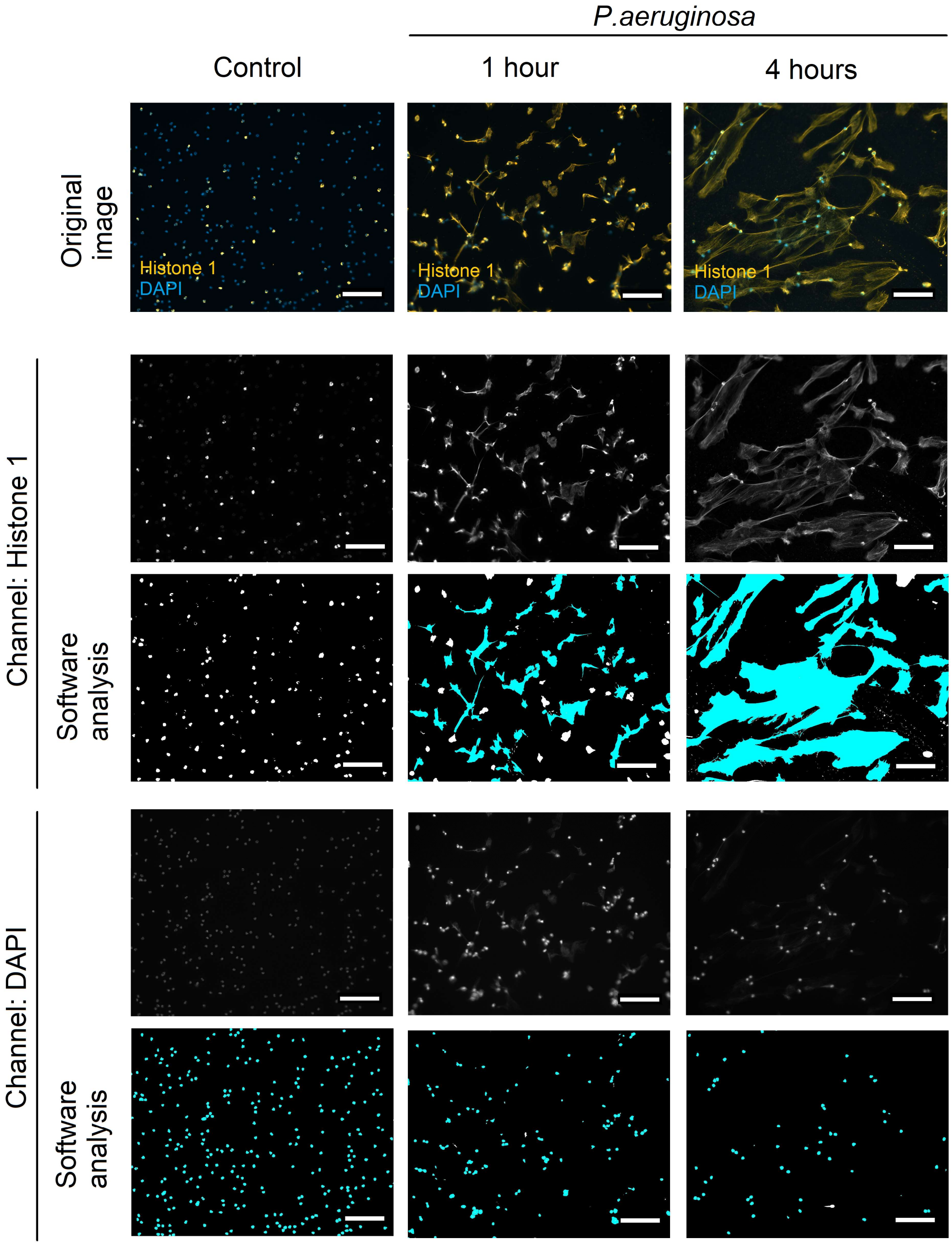

Figure S2. Example analysis of NET area using ImageJ software. Scale bar 65 μm.
